# Lost in Translation: Why Biologic Therapies for Intervertebral Disc Degeneration and Low Back Pain Have Not Reached the Clinic (Yet): Bridging Biology, Pain Phenotyping, and Trial Design

**DOI:** 10.1002/jsp2.70187

**Published:** 2026-07-03

**Authors:** Luca Ambrosio, Jordy Schol, Clara Ruiz‐Fernandez, Vincenzo Denaro, Gianluca Vadalà, Daisuke Sakai

**Affiliations:** ^1^ Laboratory for Regenerative Orthopaedics Operative Research Unit of Orthopaedic and Trauma Surgery, Fondazione Policlinico Universitario Campus Bio‐Medico Rome Italy; ^2^ Research Unit of Orthopaedic and Trauma Surgery, Departmental Faculty of Medicine and Surgery Università Campus Bio‐Medico di Roma Rome Italy; ^3^ Department of Orthopaedic Surgery Tokai University School of Medicine Isehara Japan; ^4^ Center for Musculoskeletal Innovative Research and Advancement (C‐MiRA) Tokai University Graduate School Isehara Japan

**Keywords:** clinical trial, disc degeneration, low back pain, pain, regeneration, regenerative medicine, spine, translational medicine

## Abstract

**Background:**

Biologic therapies for intervertebral disc degeneration (IDD) have shown strong preclinical promise, yet clinical translation for discogenic low back pain (LBP) remains limited. This gap reflects the inconsistent relationship between degeneration and pain, compounded by heterogeneous patient populations, insufficient phenotyping, and inadequate selection of truly discogenic LBP patients in current trials. Emerging evidence indicates that IDD and LBP comprise distinct biological endotypes, requiring matched therapeutic strategies, or “theratypes”, rather than uniform approaches.

**Methods:**

In this narrative review, we critically examined the current evidence on biologic therapies for IDD, focusing on the translational gap between preclinical success and clinical outcomes. We analyzed key methodological and biological limitations, including patient heterogeneity, pain misclassification, inadequate phenotyping, and suboptimal trial design. Additionally, we reviewed emerging concepts such as disease phenotyping, mechanism‐based patient selection, and stage‐adapted therapeutic approaches.

**Discussion:**

Multiple barriers hinder clinical translation of biologic therapies for IDD, including incomplete understanding of disc biology, limited intrinsic regenerative capacity, and a hostile tissue microenvironment. Preclinical models frequently fail to replicate the complexity of human pathology and pain, while clinical trials rely heavily on subjective outcomes and are often confounded by strong placebo effects. Furthermore, regulatory, logistical, and economic challenges limit widespread clinical adoption. Progress in the field will require a shift toward precision medicine approaches to align therapies with specific endotypes, pain phenotypes, and stages of IDD. Greater standardization, transparency, and community‐wide reporting practices are also essential to improve reproducibility.

**Conclusion:**

Advancing biologic therapies for IDD will depend on the development of more precise and biologically informed clinical strategies. Improved patient stratification, better alignment between therapeutic mechanisms and disease biology, and the adoption of standardized and clinically meaningful outcome measures are critical. Despite recent progress, key pathophysiological mechanisms remain poorly understood, underscoring the need for rigorous and multidisciplinary research efforts.

From preclinical promise to clinical translation in intervertebral disc degeneration (IDD). Robust preclinical evidence supports a range of biologic therapies, with consistent improvements in imaging, extracellular matrix composition, inflammation, and behavior. However, a substantial translational gap persists, driven by key challenges including the discordance between IDD and pain, patient heterogeneity, inadequate phenotyping, placebo effects, limitations of preclinical models, hostile disc microenvironment, and practical/clinical trial barriers. A precision medicine framework is crucial to overcome these limitations, based on improved patient stratification, definition of molecular and clinical endotypes linked to targeted therapies, integration of advanced biomarkers, development of more sensitive outcome measures, and optimization of clinical trial design, ultimately aiming to enable successful clinical translation.

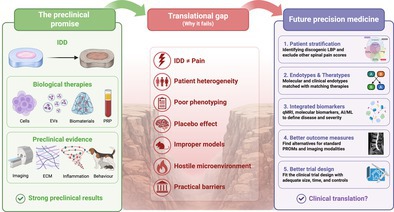

## 1 Introduction

Low back pain (LBP) is the leading cause of disability worldwide, imposing substantial social and economic burdens and affecting more than 600 million individuals globally. According to the Global Burden of Disease 2021 study [1], the prevalence of LBP is projected to increase by 36.4% during the next 25 years, with the most pronounced rises expected in Asia and Africa, largely driven by population growth. This anticipated surge, together with progressive population aging, will further amplify its socioeconomic impact, which is already comparable to that of cardiovascular diseases, cancer, and major mental health disorders [2].

Although most episodes of LBP are classified as “nonspecific”, intervertebral disc degeneration (IDD) is frequently implicated in discogenic LBP and considered a major contributing factor. In the context of IDD, the intervertebral disc (IVD) undergoes progressive cell loss and senescence, extracellular matrix (ECM) degradation with dehydration and disc height reduction, as well as pronounced neoangiogenesis and neoinnervation, immune cell infiltration, and deterioration of native biomechanical properties [3]. Despite these structural alterations, a substantial proportion of individuals with disc abnormalities remain asymptomatic [4]. While most pain flares resolve spontaneously in symptomatic cohorts, approximately 5%–10% of patients develop chronic LBP, the management of which remains challenging, as no conservative or surgical strategies directly target the underlying pathophysiological processes [3].

Over the past decades, several biologic therapies have been investigated in both preclinical and clinical settings, with the putative aim of regenerating the degenerative IVDs or, at minimum, inducing local reparative processes presumingly associated with alleviation of discogenic LBP. These approaches include cell‐based therapies (particularly mesenchymal stromal cells [MSCs]), platelet‐rich plasma (PRP), extracellular vesicles (EVs), biomaterials, growth factors, gene therapy, as well as their various combinations [3, 5, 6]. Although many of these strategies have demonstrated encouraging “regenerative” outcomes, translation from preclinical investigations to clinical trials has been inconsistent and limited to a small number of products. Nonetheless, despite a generally favorable safety profile, the application of these biologic therapies at the bedside has generally yielded only modest improvements in pain and function, with no consistent demonstration of structural repair on imaging [7]. At present, the disconnect between preclinical promise and clinical reality is tempering enthusiasm for biologics in spine care and underscores the need for a critical appraisal of the factors underlying the stalled translational pipeline.

Rather than revisiting the current literature on biologics (which has been extensively reviewed elsewhere [3, 5, 6, 7]), this study focuses on the determinants of their limited clinical translation. In particular, we examine the potential roles of patient selection, mechanistic targeting, and alignment between biological effects and clinical endpoints. Through systematic mapping of the biologic therapy landscape and thematic analysis of key failure drivers, we aim to provide an evidence‐based assessment of current translational barriers. Building on this analysis, we propose a failure‐informed roadmap that emphasizes refined patient stratification, improved concordance among mechanisms, models, and endpoints, optimization of clinical trial design, and clearer definition of the strategic role of biologics within spine care.

This review is therefore positioned not as further advocacy for biologic therapies, but as a critical evaluation of the factors that have thus far limited their clinical impact, with the aim of fostering more rigorous, evidence‐based research and advancing precision medicine–oriented approaches for LBP.

## 2 The Translational Gap: Missed Opportunities and Unmet Needs

### 2.1 IDD ≠ LBP

Beyond microenvironmental complexity and multifactorial pathophysiology, IDD poses a unique translational challenge: structural degeneration of the IVD often occurs without concordant symptoms, with LBP being directly caused by IDD solely in a fraction of patients [3]. Imaging studies show that features of lumbar IDD are common in asymptomatic individuals, increasing with age from roughly 37%–65% in subjects in their twenties until becoming nearly ubiquitous in older populations (over 96% in adults aged more than 70 years) [4, 8, 9]. These observations suggest that IDD often represents an age‐related structural phenotype rather than a direct proxy for pain, or in the words of Hammoor and colleagues [3], “IDD is an inevitable consequence of living”.

**FIGURE 1:**
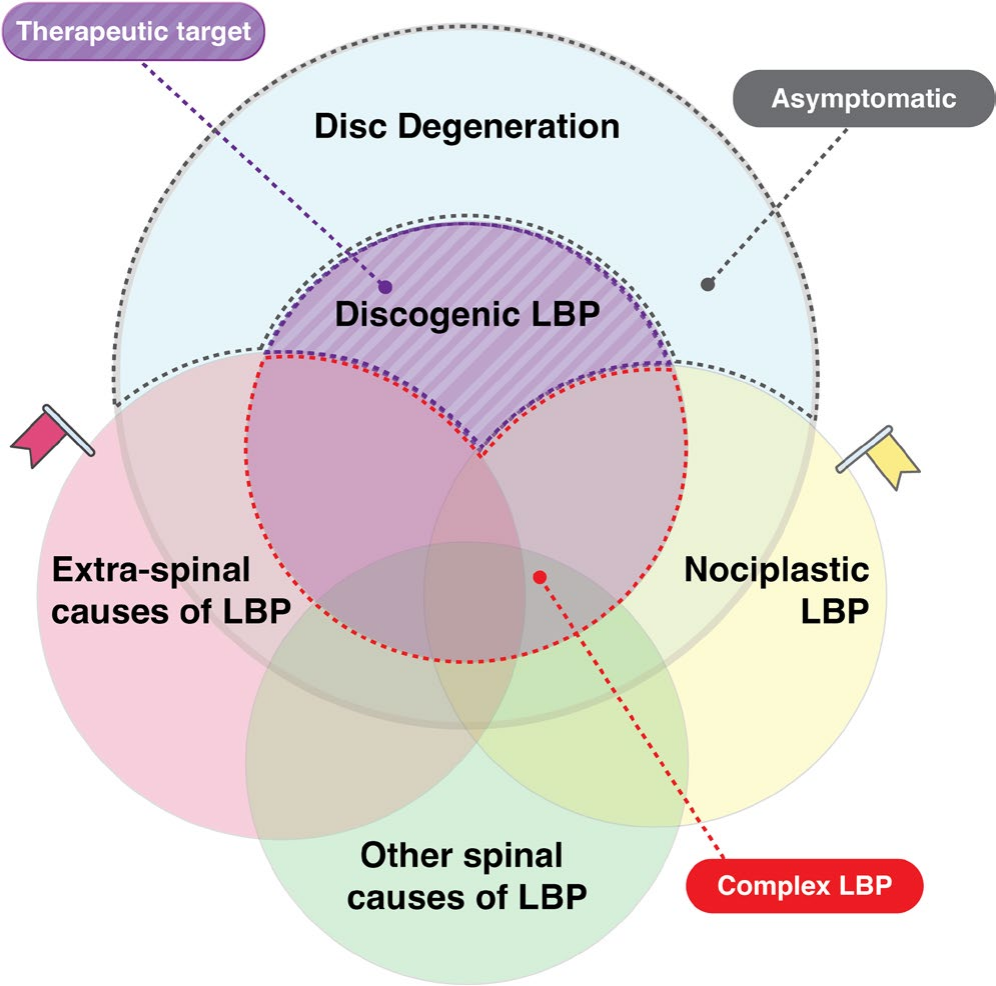
Conceptual framework of IDD and LBP phenotypes. LBP arises from multiple overlapping domains, including discogenic LBP, extra‐spinal causes (including red flags requiring urgent identification), other spinal causes (e.g., muscle spasm, facet arthropathy), and nociplastic pain (modulated by psychosocial factors, i.e., yellow flags). The subset of patients with concordant IDD and discogenic LBP (striped area) represents the most appropriate target for disc biologic therapies, whereas a large proportion of individuals with IDD remain asymptomatic (gray dashed area). In clinical practice, these domains frequently overlap, giving rise to complex LBP (red dashed area), which warrants a multidisciplinary assessment. Abbreviations: IDD, intervertebral disc degeneration; LBP, low back pain. Created with BioRender.com.

Conversely, LBP is primarily defined clinically rather than etiologically. According to the World Health Organization [10], LBP refers to pain localized between the lower margin of the twelfth rib and the gluteal fold. Within this broad anatomical definition, most cases are classified as “non‐specific” because a precise structural cause cannot be reliably identified, and many episodes resolve before a pathoanatomical diagnosis can be formulated [11]. Numerous spinal and extra‐spinal conditions can generate pain in this region, with the latter including genitourinary, gastrointestinal, and gynecological diseases (among others), and occasionally life‐threatening disorders, such as aortic aneurysm, malignancy, and infection [12]. On the other hand, spinal causes of LBP do not necessarily originate from the IVD, with alternative aetiologies including muscle spasms, fascial and ligament strains, facet joint arthropathy, and sacroiliac dysfunction [13]. Therefore, only a subset of patients experience pain that originates primarily from the IVD itself (commonly referred to as discogenic LBP), estimated between 20% and 40% of all LBP cases according to clinical and discographic assessments [14, 15, 16, 17] (Figure 1).

Despite the inconsistent concordance between IDD and LBP, an epistemologically needed, reductionist assumption has dominated the preclinical and translational literature: if degenerated IVDs are “the disease”, then regenerating or repairing the IVD tissue should relieve pain. On this path, extensive preclinical investigations using in vitro, ex vivo, and in vivo models have demonstrated biochemical, histological, and radiological repair following biological interventions targeting different pathomechanisms [18, 19, 20, 21, 22, 23]. However, pain—arguably the most clinically relevant endpoint—remains inconsistently evaluated, and the extent to which experimental models are able to reproduce the complex pain phenotype observed in humans is unclear [24, 25].

### 2.2 The Myth of a Single Pain Generator: Deconstructing Discogenic LBP

While patient heterogeneity represents one major challenge for regenerative disc therapies, an equally important limitation lies in the incomplete characterization of discogenic LBP itself as a therapeutic target. Pain in patients with lumbar IDD rarely represents a single, well‐defined biological entity. Instead, it typically emerges from the interaction of multiple mechanisms, including nociceptive, neuropathic, and centrally mediated components. In many patients, these mechanisms often coexist and dynamically evolve over time, making it difficult to ascribe symptoms to a single structural source [26, 27] (Table 1).

**TABLE 1:** Mechanistic classification of LBP: Clinical features, drivers, and diagnostic markers with implications for biologic therapies.

Pain type	Primary mechanisms	Key drivers	Clinical features	Diagnostics/biomarkers	Biologic therapy relevance
Nociceptive	Peripheral nociceptor activation from disc injury and inflammation	Annular fissures, ECM loss, neo‐innervation, cytokines, mechanical stress, CEP damage, neovascularization, immune activation	Localized axial pain, mechanical, worsens with loading or movement	MRI (HIZ, Modic changes), discography, localized QST, cytokines (e.g., IL‐1β, TNF‐α, IL‐6)	Strong target
Neuropathic	Nerve root lesion or inflammation with ectopic signaling and neuroinflammation	Disc herniation, nerve compression, DRG sensitization, neuropeptides (e.g., NGF, BDNF)	Radicular, dermatomal, burning or shooting sensations, neurologic deficits	Imaging, NCS (e.g., EMG), dermatomal QST, neuropathic pain questionnaires	Limited target Likely needs decompression or nerve‐ targeted treatments
Nociplastic	Central sensitization with enhanced spinal and supraspinal excitability	CNS hyperexcitability, reduced descending inhibition, psychosocial factors	Widespread or disproportionate pain, hypersensitivity, allodynia, temporal variability, issues with fatigue, sleep, and mood	QST widespread changes; CPM impairment; PROMs (e.g., BDI, PCS, DASS‐21), fMRI or EEG	Poor target Likely needs central and multidisciplinary approaches

Abbreviations: BDI: Beck depression inventory; BDNF: brain‐derived neurotrophic factor; CEP: cartilaginous endplate; CNS: central nervous system; CPM: conditioned pain modulation; DASS‐21: Depression anxiety and stress scale‐21; DRG: dorsal root ganglion; ECM: extracellular matrix; EEG: electroencephalography; EMG: electromyography; fMRI: functional magnetic resonance imaging; HIZ: high‐intensity zone; IL: interleukin; MRI: magnetic resonance imaging; NCS: nerve conduction studies; NGF: nerve growth factor; PCS: Pain catastrophizing scale; PROMs: patient‐reported outcome measures; QST: quantitative sensory testing; TNF‐α: tumor necrosis factor alpha.

Nociceptive pain has traditionally been attributed to the mechanical or inflammatory stimulation of nociceptors following structural disruption of the annulus fibrosus (AF), such as annular fissures, which may promote local neo‐innervation and activation of the sinuvertebral nerve [28]. Conversely, neuropathic pain has classically been associated with nerve root compression and/or neuroinflammatory changes in the context of IDD [27]. However, both clinical observations and emerging molecular evidence suggest that nociceptive and neuropathic mechanisms rarely exist as separate entities. Rather, they appear to intersect along a complex biological spectrum involving pro‐inflammatory cytokines (e.g., interleukin [IL]‐1β, IL‐6, IL‐17, tumor necrosis factor [TNF]‐α, interferon [IFN]‐γ), neurotrophins (e.g., nerve growth factor [NGF], brain‐derived neurotrophic factor [BDNF]), neuropeptides (e.g., substance P and calcitonin gene‐related peptide [CGRP]), and pro‐angiogenic mediators (e.g., vascular endothelial growth factor [VEGF], platelet‐derived growth factor [PDGF]). Additional contributors, including local and systemic microbiota‐related factors, have also been proposed as potential modulators of discogenic inflammation and nociception [16, 29, 30, 31]. While several biologic therapies for IDD aim to modulate these key pathways [3, 22, 32, 33, 34, 35, 36], it is unlikely that any single intervention can simultaneously address the full spectrum of mechanisms involved in discogenic LBP. Moreover, the specific molecular drivers of pain may vary substantially among patients and even evolve throughout the course of IDD. Consequently, a therapy targeting a limited subset of pathways may not necessarily coincide with the dominant mechanisms active in an individual patient at the time of treatment and throughout the follow‐up.

The challenge is further compounded by the multifactorial nature of LBP. Contemporary understanding of chronic LBP is grounded in a biopsychosocial framework in which nociceptive and neuropathic inputs interact with central nervous system processing and psychosocial modifiers such as fear‐avoidance behavior, kinesiophobia, psychosocial distress, and occupational factors [2, 37, 38, 39, 40, 41, 42] (Table 1). Over time, central sensitization may amplify or sustain pain independently of the original peripheral generator, progressively decoupling the patient's pain experience from the underlying structural pathology, and posing the basis for the development of chronic LBP [43, 44].

In this context, discogenic LBP may be further complicated by the presence of nociplastic pain, defined as pain arising from altered nociception despite the absence of clear evidence of active tissue damage or a distinct lesion of the somatosensory system. Clinically, it is often characterized by pain that appears disproportionate to structural findings, fluctuates unpredictably, and is accompanied by widespread hypersensitivity to both musculoskeletal and non‐musculoskeletal stimuli [45]. Interestingly, features consistent with nociplastic pain have been increasingly reported in patients with chronic LBP [46], who represent, nonetheless, the main population enrolled in clinical trials evaluating biologic therapies for IDD [6, 7]. In these individuals, LBP is predominantly driven by dysregulated pain‐processing pathways involving both the peripheral and central nervous systems [45], with possibly coincidental or slightly symptomatic IDD. Furthermore, the renowned tendency toward overmedicalization, increased risk of adverse events, and limited effectiveness of conventional interventions make this subgroup particularly susceptible to poor outcomes with intradiscal biologic therapies [46].

Despite these nuances, patient selection in many clinical trials evaluating disc biologic therapies remains largely based on the coexistence of chronic LBP and contingent radiological evidence of IDD, commonly assessed through imaging markers such as disc height loss or nucleus pulposus (NP) dehydration at magnetic resonance imaging (MRI) [6, 7, 47]. In several published studies, attempts to confirm a discogenic pain source are limited or absent. When diagnostic strategies are employed, they rely either on clinical symptom patterns (which remain subjective and poorly specific) or provocative discography (PD). Nonetheless, PD is characterized by well‐documented methodological controversies and potential harms, including reports of accelerated IDD, lumbar disc herniation, allergic reaction to iodine‐based contrast agents, and increased risk of subsequent surgery [16, 48, 49].

Importantly, most trials have not systematically triaged biopsychosocial modifiers or used robust phenotyping to isolate patients whose pain is dominantly discogenic and minimally influenced by central or psychosocial factors, therefore introducing confounding [6, 7, 47]. Consequently, the resulting study populations are highly heterogeneous: the proportion of patients with truly dominant discogenic LBP might be relatively small, and an even smaller subgroup may include the ideal candidates for targeted biologic interventions. Under these circumstances, therapeutic effects may be diluted by phenotypic heterogeneity, potentially obscuring meaningful outcomes.

## 3 Rethinking IDD Biology: Mechanistic Uncertainty and Therapeutic Limits

Despite extensive preclinical research on biologic strategies for IDD, important uncertainties remain regarding the mechanisms these therapies aim to target. A central issue is the frequent conflation of repair and regeneration. Many biologic interventions are described as regenerative, yet their effects often resemble partial repair or mitigation of tissue damage rather than true restoration of disc structure and function. This distinction is critical because the adult IVD has limited intrinsic regenerative capacity, particularly at advanced stages of IDD.

For example, the formation of cartilage‐like tissue within the IVD does not necessarily restore the specialized NP matrix, which contains substantially higher proteoglycan‐to–type II collagen ratios than articular cartilage [50, 51]. Although MSCs can differentiate toward a chondrogenic phenotype, this does not ensure production of the unique ECM required to restore native tissue architecture and biomechanical function. Such approaches may nevertheless provide clinical benefit by slowing IDD progression or replacing the degenerative tissue with a less catabolic or less nociceptive phenotype [52]. However, these outcomes are more accurately described as repair or functional replacement rather than true regeneration.

Another unresolved question is which structural alterations within the IVD remain biologically reversible. While early biochemical changes in IDD may retain some degree of plasticity, later structural abnormalities such as annular fissures or cartilaginous endplate (CEP) sclerosis likely represent largely irreversible pathologies. During normal development and maturation, increasing mechanical demands are accommodated through coordinated alignment of cells and ECM components regulated by biochemical and mechanical signals [53]. Degeneration disrupts this organization and alters the native mechanical environment, generating aberrant cues that may impair cellular responses and tissue remodeling [54]. Whether biologic therapies can overcome these chronic mechanical disturbances remains uncertain. In parallel, aging and IDD are associated with increased cellular senescence and apoptosis, resulting in both reduced cell numbers and a higher proportion of metabolically unresponsive cells [53, 55, 56, 57]. As a consequence, biologic agents that depend on resident cell responsiveness, such as growth factors, PRP, or EV‐based therapies, may have limited efficacy in advanced IDD due to the reduced availability of viable target cells.

The nutritional microenvironment of the IVD represents an additional constraint [58, 59, 60]. As the largest avascular tissue in the body, the disc relies on diffusion through the CEPs to supply oxygen and glucose while removing metabolic waste. IDD compromises this transport system through CEP calcification, reduced CEP permeability, and declining proteoglycan content, collectively limiting nutrient diffusion and promoting an acidic microenvironment [55, 61, 62]. Computational and patient‐specific diffusion modeling suggest that nutrient availability in many degenerated IVDs may already approach the threshold required to sustain cell viability. Under these conditions, therapies that increase cell density or metabolic activity may further elevate demand within an already constrained niche [63, 64, 65]. While the degenerative IVD reportedly contains approximately 1–2 million cells/mL, the number of cells delivered in clinical studies varies widely, ranging from near‐physiological levels to more than 60‐fold higher concentrations [7, 47]. Such variability raises critical questions regarding cell survival in the nutrient‐limited IVD environment and the potential biological consequences of widespread cell death, including the release of intracellular byproducts following starvation or apoptosis. Despite these concerns, these aspects remain poorly characterized in the available literature.

Timing may represent another critical yet scarcely defined variable. Clinically, patients with chronic LBP rarely present during early degenerative stages, meaning structural damage is often already established at the time of treatment. A therapeutic window likely exists in which biologic interventions can meaningfully influence disease progression. Early stages, characterized by viable cell populations and partially preserved ECM architecture, may remain responsive to biologic modulation, whereas advanced IDD marked by fibrosis, structural collapse, and mechanical instability likely exceeds the reparative capacity of such approaches. Consequently, the mechanistic basis of many biologic therapies for IDD remains incompletely understood [66], highlighting the need to better define the limits of tissue reversibility, the influence of the disc microenvironment, and the relationship between structural modification and clinical outcomes. These mechanistic uncertainties complicate the rational design of biologic therapies and may partly explain why promising preclinical findings have not consistently translated into clinical success.

## 4 Modeling the Wrong Disease? Limits of Preclinical IDD Systems

A central limitation of IDD preclinical research is species‐specific disc biology, which can profoundly shape degeneration kinetics, repair capacity, and the success of investigational therapies. Many widely used small animal models, like mice or rats, offer established experimental control and behavioral testing options, yet typically retain notochordal cells into adulthood and differ from humans in IVD size, biomechanics, and transport properties, factors that may limit direct extrapolation to adult human IDD [16, 67, 68]. Rabbits, commonly used for puncture‐induced IDD because of procedural accessibility and disc size, likewise retain notochordal cells and may exhibit relatively robust intrinsic healing, making them valuable for early‐stage assessment, but are likely too optimistic for translation [69]. In contrast, large‐animal models (e.g., sheep, goat) better approximate human IVD dimensions, CEP morphology, and mechanical environment, supporting surgical reproducibility and implant or biomaterial testing, albeit with greater cost, longer time courses, and higher regulatory burden [70, 71]. Likewise, porcine models can provide relevant loading environments and biochemical similarities, yet notochordal cell retention may persist depending on breed, and practical constraints often confine their use to later‐stage validation [16, 23, 72]. Importantly, canine models illustrate how species selection can either hinder or enhance clinical relevance: non‐chondrodystrophic breeds maintain a notochordal‐rich, gelatinous NP longer and are less representative of adult human IDD [73], whereas chondrodystrophic breeds develop more human‐like, age‐associated IDD and are increasingly viewed as a clinically relevant intermediate‐sized model, particularly for questions involving pain‐related outcomes [74, 75]. Nevertheless, small animal models remain widely used due to cost efficiency, regulatory expectations, and the requirement for efficacy data in these systems before progression to large animal studies, despite their recognized limitations.

Even when degeneration is successfully induced, many models reproduce IDD without faithfully modeling discogenic LBP, not differently from their human counterpart. In discogenic pain research specifically, most studies are performed in rodents, supported by established behavioral testing batteries; however, their translational potential to humans remains limited, and conventional imaging, although a gold standard for structural change, cannot reliably identify disc‐related pain [43, 76].

A related challenge is that widely used induction paradigms, particularly puncture‐based injury models, often create an acute, externally applied (“outside‐in”) insult that differs from the chronic, spontaneous (“inside‐out”) trajectory often implicated clinically. Such models may disproportionately emphasize the role of the AF and NP while underrepresenting contributions from CEPs, vertebral bone, and posterior elements, tissues that can be relevant to human IDD and pain biology. Therefore, they should be better regarded as models of acute injury rather than chronic degeneration. Methodological heterogeneity (e.g., needle gauge, depth, dwell time, number and site of punctures, among others) further complicates cross‐study comparison and reproducibility [23].

Finally, many preclinical studies foreground short‐term outcomes and surrogate readouts (e.g., histology scores, MRI, or biomarker panels) while providing limited evidence for durability and functional relevance. Structural improvement does not necessarily imply restoration of disc mechanical performance, stabilization of the motion segment, or sustained modulation of nociceptive signaling [16]. Recent work emphasizes that behavioral assays are informative but can be confounded by stress, anxiety, and environmental conditions, reinforcing the need for integrated outcome frameworks that combine structure, biology, biomechanics, and pain‐related function over clinically meaningful timeframes [23, 67]. Collectively, these limitations argue for a stricter alignment among model choice, disease mechanism, and endpoint selection, and for prioritizing disease models and study designs that better capture human‐relevant disc biology, pain‐relevant pathways, and durable functional outcomes rather than short‐term structural surrogates alone.

## 5 Limitations in Current Clinical Trial Design in IDD: Misalignment and Missed Outcomes

As with other investigational medical products, biologic therapies targeting IDD are expected to progress through the conventional translational pathway and demonstrate safety and efficacy across Phase 1–3 clinical trials before being adopted into routine clinical practice. Yet the likelihood of successful translation is notoriously low. It is estimated that approximately 95% of drug candidates entering human trials ultimately fail, with nearly half of these failures occurring during already advanced Phase 3 studies. Consequently, only a very small proportion of experimental therapies successfully translate from bench to bedside, approximately 0.1% [77]. Notably, while the vast majority of biologic therapies investigated in vitro and in preclinical models of IDD have never reached clinical testing, many of the products that have entered clinical practice have been evaluated primarily in non‐comparative cohort studies rather than in rigorous randomized controlled trials (RCTs).

To date, MSCs represent the biologic therapy supported by the largest body of clinical evidence, with 5 RCTs published to date (including Phase 2a, Phase 2b, and Phase 3 studies) [78, 79, 80, 81, 82]. Early uncontrolled reports consistently described substantial improvements in LBP severity following treatment, occasionally accompanied by modest structural changes on imaging. However, these encouraging results were markedly attenuated in randomized controlled settings. In several RCTs, the differences between MSC‐treated and sham‐treated groups were small, and although statistical significance was occasionally achieved, the observed improvements frequently failed to surpass the minimal clinically important difference (MCID) for key outcome measures [6]. Regarding other cell types, the recently published Phase 1/2 DiscGenics trial did not show any significant differences between the treatment (high‐ and low‐dose NP progenitor cells) and control groups (vehicle only and saline) [83]. Likewise, the few RCTs investigating the efficacy of intradiscal PRP were characterized by low sample sizes and often demonstrated no significant differences compared to control groups [84, 85, 86]. Two NP graft strategies, NOVOCART Disc and VIA Disc NP, have also been evaluated in RCTs. NOVOCART Disc consists of a two‐stage procedure in which autologous NP cells are first harvested during discectomy for lumbar disc herniation, expanded, and subsequently injected upon resuspension in a matrix composed of human albumin, hyaluronic acid, and a cross‐linking agent. Although treatment resulted in modest reductions of pain scores, these improvements were transient and not accompanied by meaningful structural changes on imaging. Consequently, clinical development of this approach was ultimately discontinued [52, 87, 88]. Conversely, the VIA Disc NP, an injectable human NP allograft, was evaluated in an RCT compared with saline. Both groups demonstrated clinically meaningful improvements in Visual Analogue Scale (VAS) and Oswestry Disability Index (ODI) scores, with a significantly higher responder rate (and number of adverse events) in the VIA Disc NP group [89].

Despite the scarcity of published high‐level clinical evidence, international trial registries such as ClinicalTrials.gov list dozens of protocols investigating biologic therapies for IDD. In a previous analysis from our group, we reported that only 26.9% of registered clinical trials had ultimately resulted in a peer‐reviewed publication [90]. Despite the recent publication of four additional RCTs [79, 80, 81, 83], the proportion of trials failing to reach completion or publication remains substantial. From a clinical trial design perspective, this may reflect several factors.

First, neglecting the inconsistent association between LBP and IDD in most clinical trials creates a substantial risk of selection bias. Many studies enroll patients with imaging evidence of IDD without definitively establishing the discogenic origin of pain, thereby potentially including individuals with non‐discogenic LBP and incidental IDD findings. As a result, the likelihood of enrolling patients whose symptoms arise from alternative pain generators despite concomitant IDD is considerable, introducing significant confounding. In this context, the administration of intradiscal biologic therapies may lead to biased estimates of treatment efficacy, as the targeted tissue pathology may not represent the true source of pain.

Second, difficulties in patient recruitment and high attrition rates may further compromise study feasibility and statistical validity, possibly leading to early termination or critical lack of statistical power. Among published trials, loss to follow‐up rates range from 9% [52] to 34% [79], posing a substantial threat to the internal validity of reported outcomes. Beyond the burden imposed on participants (often including repeated imaging examinations, blood sampling, and lengthy questionnaire completion), trial attrition may also be influenced by placebo and nocebo effects [91]. These phenomena are particularly relevant in populations with chronic LBP, where, as mentioned, psychological distress and anxiety are common, especially in patients with nociplastic pain features [46].

With regard to study endpoints, most trials rely primarily on patient‐reported outcome measures (PROMs), including pain intensity (e.g., VAS), disability (e.g., ODI), and quality of life (e.g., EuroQol [EQ]‐5D and Short Form [SF]‐36). Structural outcomes, including disc height and NP hydration on MRI, are frequently assessed, though they are often inadequately reported [6, 7]. PROMs are inherently subjective and susceptible to multiple psychological and contextual influences, but remain necessary to determine treatment success or failure in a real‐world scenario [92]. At present, no validated surrogate endpoint of pain exists. Although approaches such as quantitative sensory testing, neuroimaging, or inflammatory biomarkers have been explored, none have demonstrated sufficient validity or reliability to replace PROMs of pain in clinical trials [93]. In the IDD field, imaging parameters have long been proposed as surrogate outcomes, yet clinical trials have reported only sporadic cases of disc height preservation or increased disc hydration [6, 7]. Whether such structural changes can be reliably detected (or even occur) within the relatively short duration of most clinical studies remains uncertain. For example, modeling studies suggest that, even under optimistic assumptions of a linear relationship between injected cell number and glycosaminoglycan (GAG) synthesis, administration of 10 × 10^6^ cells would restore only about 65% of the GAG content of a Pfirrmann grade 2 IVD over approximately ten years when applied to a Pfirrmann grade 3 IVD [63]. Such prolonged timeframes are fundamentally misaligned with the duration of most clinical trials and pose a significant translational constraint, apart from having doubtful clinical relevance [66]. Taken together, these observations illustrate a fundamental paradox: clinical trials in IDD rely on subjective measures to evaluate therapies whose objective structural effects are not necessarily associated with their outcomes.

Another methodological aspect that complicates the interpretation of the results of these clinical trials is the use of composite and responder‐based endpoints. In addition to (or instead of) reporting mean changes and standard deviations of pain or disability scores, several studies present outcomes as the categories of participants reporting a certain score range, the proportion of patients achieving predefined thresholds of improvement, or the combination of multiple outcomes into composite measures [78, 79, 80, 89]. While these approaches may increase statistical power and provide clinically interpretable categories of therapeutic response, they may also complicate the interpretation of treatment effects, particularly when the overall mean differences between treatment groups are small [94]. Methodological studies have highlighted that composite endpoints may amplify the apparent magnitude of benefit when driven by less clinically meaningful components or when thresholds are selected post hoc [95, 96]. In conditions such as chronic LBP, where placebo responses are substantial and treatment effects are often modest, such analytical strategies may contribute to apparently positive findings despite limited absolute differences between intervention and control groups.

Taken together, the most critical limitation is inadequate patient selection, as enrolling heterogeneous populations without confirming a dominant discogenic pain source likely dilutes mechanism‐specific treatment effects. This is compounded by reliance on subjective PROMs without validated objective correlates, particularly with the strong placebo responses, and by the mismatch between slow biological repair and short trial durations. In addition, incomplete reporting, including the use of composite endpoints and limited structural outcome data, further limits interpretability. Addressing these factors collectively is likely to yield the greatest improvements in trial validity and near‐term translational potential.

## 6 Barriers Beyond Biology: Practical, Regulatory, and Economic Hurdles

Beyond biological and clinical uncertainties, the translation of disc biologic therapies is also constrained by substantial practical, regulatory, and economic barriers. Even when promising signals emerge from preclinical studies or early‐phase clinical trials, these challenges can significantly delay or prevent broader clinical adoption [90, 91, 92, 93, 94, 95, 96, 97].

Manufacturing complexity represents one of the most significant practical obstacles. Many biologic approaches, particularly cell‐based or cell‐derived therapies, require highly specialized production processes that must meet strict standards for sterility, consistency, and quality control [97, 98, 99]. Autologous therapies typically involve multistep workflows including cell harvesting, expansion, characterization, and reimplantation, each introducing logistical challenges, variability, and costs. In addition, the potency of these products may be affected by patient factors, as individuals with chronic LBP are often older and present with comorbidities that may impair the regenerative capacity of harvested cells [100, 101]. On the other hand, allogeneic approaches may offer greater scalability but introduce additional concerns related to immunogenicity, storage conditions, and long‐term safety [97].

Regulatory pathways for biologic therapies are also more complex than those for conventional pharmaceuticals or medical devices. Many disc biologic interventions fall within hybrid regulatory categories such as advanced therapy medicinal products or combination products [98, 102]. These classifications require evaluation of both the biologic component and the delivery system, which may include injectable carriers, biomaterial scaffolds, or surgical transplantation techniques. Navigating these frameworks is often time‐consuming and costly, particularly when long‐term safety monitoring is required, and clinically meaningful endpoints remain difficult to define.

Risk–benefit considerations further complicate regulatory and clinical adoption. Discogenic LBP is not a life‐threatening condition, and symptom severity often fluctuates over time. Consequently, regulatory agencies and stakeholders typically require strong evidence of safety and durable clinical benefit before accepting invasive or costly biologic interventions. Even relatively small risks related to immune reactions or unintended tissue responses may be viewed cautiously when established treatments such as rehabilitation, pharmacologic therapy, or surgery remain available. Demonstrating therapeutic efficacy is also challenging, as structural imaging changes do not consistently correlate with symptoms, while patient‐reported outcomes remain subjective and highly susceptible to placebo effects [103, 104, 105].

Economic considerations add another layer of complexity. As biologic therapies frequently involve high development and manufacturing costs (translating into substantial treatment prices), healthcare systems and payers might be reluctant to reimburse such treatments without convincing evidence of long‐term cost‐effectiveness. Such a challenge is further amplified by the modest, delayed, or difficult‐to‐quantify clinical benefits assessed with conventional outcome measures. Furthermore, the absence of clear reimbursement pathways introduces uncertainty regarding commercial viability and may limit investments from industry and private stakeholders.

These considerations suggest that practical, regulatory, and economic barriers are not independent obstacles but rather downstream manifestations of unresolved biological questions and suboptimal trial design. Even when biological plausibility and early clinical promise exist, successful implementation of disc biologic therapies requires coordinated progress across scientific, regulatory, and health economic domains. Despite the large patient population and substantial market potential associated with such therapeutic tools, these challenges continue to limit the translation of regenerative medicine into widely adopted clinical pipelines.

## 7 How to Fill the Translational Gap: Lessons Learned and Precision Medicine Approaches

### 7.1 Research Focus Shifts Are Needed

Bridging the translational gap will require a deliberate re‐orientation of preclinical modeling toward chronicity, pain relevance, and patient‐matched biology, rather than continued reliance on highly standardized but biologically simplified IVD injury paradigms. First, the field should progressively shift from predominantly acute models (e.g., puncture‐driven IDD) toward progressive degeneration that better reflects the slow accumulation of ECM failure, CEP dysfunction, inflammation, and neurovascular remodeling observed clinically [106]. Here, alternative approaches such as mechanically induced degeneration under physiologic loading, IDD promoted by CEP damage, genetically predisposed models, natural aging, or spontaneous disease in companion animals (e.g., canine patients [107, 108]) may offer greater biological fidelity [74, 75, 109]. However, these models introduce trade‐offs, including increased cost, complexity, longer study duration, regulatory complexity, and may reduce experimental control and standardization.

Second, models must reflect the target patient's prevalent pathophysiological mechanisms. Therefore, careful selection of species, age, sex, and relevant comorbidities (among other factors) should align with the intended indication, enabling appropriate matching to the most suitable biologic approach. Critically, human relevance is strongly determined by the IVD microenvironment: models should examine and incorporate physiologic loading and nutrient constraints, as many interventions are otherwise evaluated under permissive conditions that fail to capture transport limitations, altered mechanotransduction, and the harsh metabolic environment of the adult human IVD [109]. A parallel shift is needed in outcomes: while structural repair remains important, greater emphasis should be placed on pain‐related and functionally anchored endpoints, including behavioral assays where appropriate, as discogenic LBP is not a simple readout of morphology and may persist despite imaging improvement. In this context, demonstrating durable modulation of nociceptive and neuroimmune pathways may provide more meaningful evidence of therapeutic efficacy. Finally, precision translation will require omics‐informed and computationally supported model interpretation: integrating transcriptomic and proteomic profiles, single‐cell atlases, and network modeling can help distinguish reversible versus entrenched pathways, identify mechanism‐specific biomarkers of response, and clarify whether an intervention drives transient repair (symptomatic or structural improvement) versus true regeneration (restoration of healthy IVD‐like tissue organization and homeostatic function) [16]. In this context, therapies should be tested across multiple degeneration stages, as interventions that succeed in early biochemical dysfunction may fail in late‐stage structural collapse, and the preclinical evidence base should explicitly map stage‐dependence rather than assuming a unified biological model of IDD [106].

### 7.2 Defining the Target Population for Disc Biologic Therapies

A key strategy to improve the interpretability of clinical trials in biologic therapies for IDD is the reduction of clinical heterogeneity through more rigorous patient selection. First, future studies should aim to identify patients with a dominant discogenic pain phenotype, rather than broadly enrolling individuals with chronic LBP and coincidental imaging findings of IDD. This process should begin with careful integration of patient history and physical examination (components that are often overlooked but remain essential for clinical phenotyping), subsequently complemented by imaging. Clinical evaluation should confirm the chronicity of LBP (≥ 3 months) and assess features typically associated with discogenic pain, including insidious onset, midline lumbar tenderness, reduced range of motion, symptom exacerbation with flexion and sitting, and absence of neurological signs or features suggestive of neuropathic pain. When other spinal causes of LBP are suspected, including sacroiliac joint dysfunction or facet joint syndrome, the use of intraarticular sacroiliac injections [110] and medial branch blocks [111], respectively, is recommended to confirm or rule out these alternative LBP sources.

In parallel, the presence of nociplastic traits should be systematically assessed and, where predominant, considered a potential exclusion criterion [13]. In this regard, the recommendations of the BACPAP consortium provide a useful framework for identifying patients with significant nociplastic components and may represent a valuable tool during trial screening [46]. Furthermore, psychosocial risk factors (commonly referred to as “yellow flags”) should be carefully evaluated, as they may strongly influence pain perception, disability, and treatment response. In patients where such factors appear to play a predominant role in symptom generation, biologic interventions may be unlikely to provide meaningful benefits, and alternative multidisciplinary approaches incorporating psychological and behavioral therapies may be more appropriate [10, 112].

To facilitate this process, screening protocols should incorporate validated PROMs assessing psychosocial domains, such as the Beck Depression Inventory (BDI), Pittsburgh Sleep Quality Index (PSQI), Depression Anxiety and Stress Scale‐21 (DASS‐21), Pain Catastrophizing Scale (PCS), Hospital Anxiety and Depression Scale (HADS), and Patient Health Questionnaire‐9 (PHQ‐9) [2, 113]. Investigators should carefully monitor these measures and consider excluding or stratifying patients who meet predefined thresholds for psychosocial distress, thereby increasing the likelihood that observed treatment effects reflect modulation of disc‐derived pain mechanisms rather than non‐IVD‐derived contributors.

Imaging‐based phenotyping should also be refined to better characterize disc structure and biological viability, moving beyond conventional grading systems toward more detailed assessments of IVD composition and integrity. Although the Pfirrmann classification [114], particularly in its more granular modified version [115], has demonstrated excellent inter‐rater agreement and reliability [116, 117], it primarily captures morphological features such as NP dehydration and disc height loss, and is therefore limited in detecting earlier biochemical changes that precede overt structural IDD.

To address these limitations, several quantitative MRI techniques have been developed over the past decade, including T1ρ, T2 mapping, T2*, diffusion‐based imaging, and glycosaminoglycan chemical exchange saturation transfer (gagCEST) [118, 119]. These novel sequences enable assessment of matrix composition and disc integrity, while CEP characterization may serve as a surrogate for nutrient transport capacity [104, 120]. Complementing these approaches, magnetic resonance spectroscopy (MRS) is emerging as a promising tool for the non‐invasive dissection of the biochemical composition of the IVD [105, 121, 122]. By detecting metabolites associated with anabolic processes (e.g., proteoglycans and collagen) as well as catabolic byproducts (e.g., lactic acid, propionic acid, and lipids), this technique may help identify early or specific degenerative changes before they become detectable by conventional structural imaging or Pfirrmann‐based grading [123, 124]. Interestingly, MRS has been shown to strongly correlate with PD, demonstrating a 100% negative predictive value in ruling out discogenic pain in non‐herniated IVDs [124]. In addition, recent studies have reported that patients displaying a predominant lipid peak on MRS exhibited higher pain and disability scores, intracellular triglyceride accumulation, and upregulation of the IL‐17 signaling axis [125]. Nevertheless, these findings remain preliminary, and the clinical utility and large‐scale validity of these advanced imaging approaches still require confirmation in well‐designed prospective studies. Along conventional MRI features of IDD, high‐intensity zones (HIZs) and Modic changes are additional imaging phenotypes often associated with discogenic LBP. HIZs, typically observed as focal hyperintense signals within the posterior AF on T2‐weighted MRI, have been linked to annular fissures [126, 127]. Similarly, Modic changes (particularly type 1) have shown a more consistent association with pain than IDD alone, likely reflecting active CEP and subchondral bone remodeling. Growing evidence suggests that these imaging features may arise from heterogeneous underlying mechanisms, including mechanical stress, inflammation, and, in some cases, low‐grade infection [60, 128]. Although several trials have considered screening for annular integrity and Modic changes, more consistent implementation of these imaging phenotypes should be encouraged.

Furthermore, nuclear imaging modalities, including Single Photon Emission/Computed Tomography (SPECT)/CT [129] and Positron Emission Tomography (PET) [130], have also demonstrated the ability to detect metabolically active CEPs and IVDs, suggesting a potential role in identifying pain‐generating segments. However, these techniques primarily reflect secondary processes such as inflammation and bone remodeling rather than disc pathology itself, are associated with substantial radiation exposure, and still remain insufficiently validated for routine diagnostic use.

Greater emphasis should be placed on integrating patient‐specific anatomical and clinical data into in silico platforms to estimate nutrient availability, metabolic constraints, and biomechanical loading conditions [62, 63, 64, 131]. Although such models are still evolving, in silico models informed by patient‐specific imaging datasets may enable robust prediction of treatment feasibility and response. Their integration into clinical trial design, particularly through artificial intelligence (AI)‐driven frameworks, represents a promising yet underexplored opportunity [132].

Systemic metabolites and signaling molecules have been increasingly linked to LBP and IDD and may serve as complementary tools for patient stratification and therapy selection. Circulating inflammatory mediators such as IL‐6, TNF‐α, and C‐reactive protein, as well as disc‐derived matrix degradation products (e.g., aggrecan fragments), have been associated with IDD severity and pain, and may support stratification of patients with active inflammatory or catabolic disc environments [133, 134].

**FIGURE 2:**
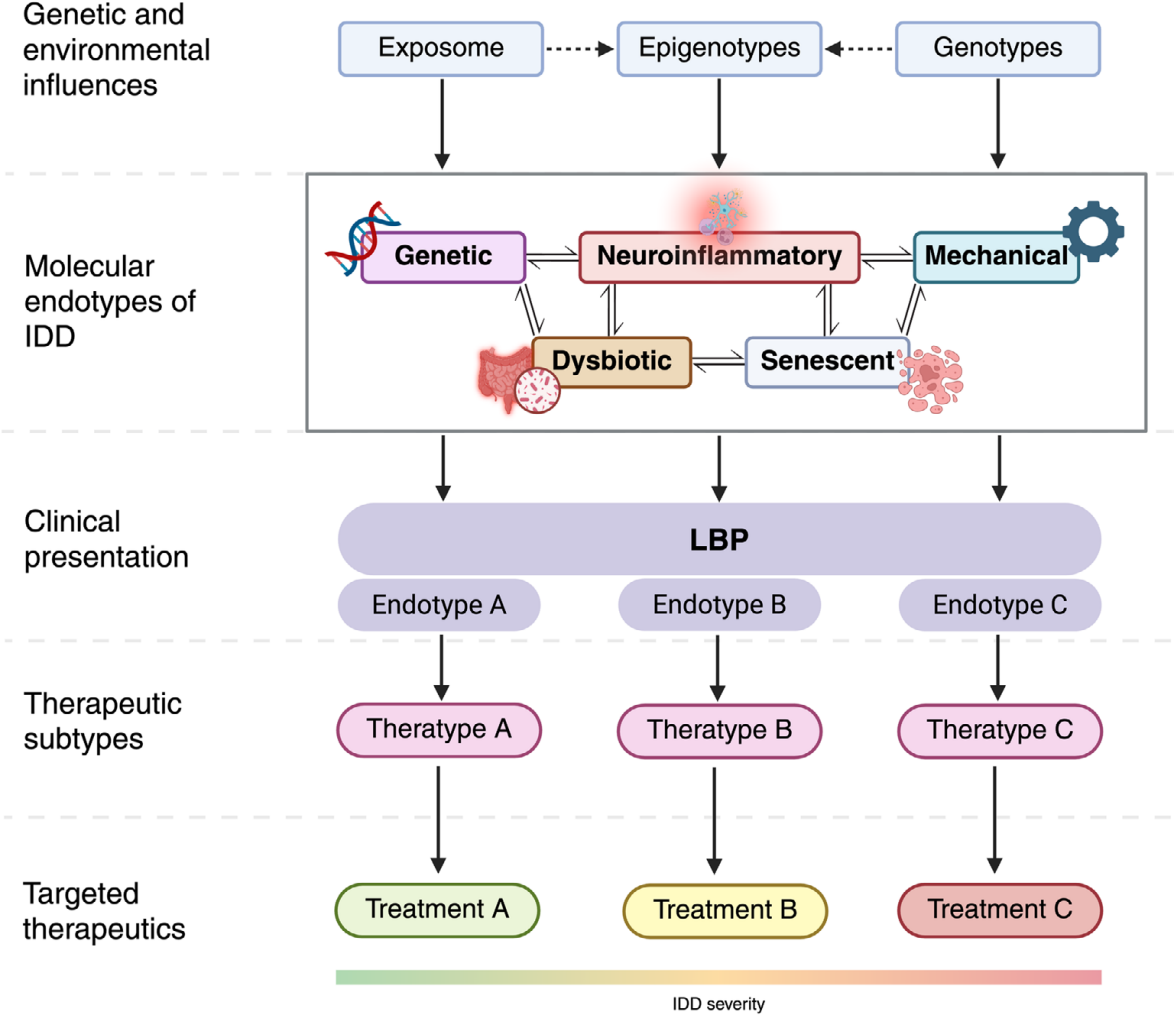
From molecular endotypes to precision therapeutics in IDD. Genetic and environmental influences, including genotype, epigenetic modifications, and exposome interact to shape distinct molecular endotypes of IDD, encompassing genetic, neuroinflammatory, mechanical, dysbiotic, and senescent pathways. These interconnected biological processes contribute to generating the clinical phenotype of discogenic LBP. Integration of multi‐omics, biomarkers, advanced imaging, and AI is expected to enable endotype‐driven classification of patients, facilitating the identification of specific therapeutic subtypes (“theratypes”). In this framework, targeted interventions are tailored to the underlying pathobiology, enabling a transition from symptom‐based management to mechanism‐based precision medicine. The gradient reflects increasing IDD severity and the corresponding shift in therapeutic strategies. Adapted from Mobasheri et al. [146]. Abbreviations: AI, artificial intelligence; IDD, intervertebral disc degeneration; LBP, low back pain. Created with BioRender.com.

Furthermore, the design of clinical trials and the choice of outcomes should be more closely aligned with the biological mechanisms of investigated therapies. A major limitation of current studies lies in the implicit assumption that a given biologic approach can be broadly applied across heterogeneous patient populations, despite substantial differences in the underlying pathomechanisms of IDD. Increasing evidence supports the existence of distinct IDD *endotypes*, potentially driven by diverse pathophysiological processes including genetic predisposition [135], metabolic dysfunction [136, 137, 138], mechanical overload [139, 140], neuroinflammation [29, 30, 141], cell senescence [142, 143], and microbiome‐related factors [144, 145]. Although these mechanisms may converge into a similar clinical phenotype, namely LBP, they may represent biologically distinct conditions requiring tailored therapeutic strategies, or “*theratypes*”. A similar conceptual shift has recently emerged in the field of osteoarthritis, where joint pain (long attributed to a uniform “wear‐and‐tear” process, not far from the generic “disc dehydration” image of IDD) has been reinterpreted through the lens of molecular endotyping (Figure 2). This paradigm change reflects the recognition that biologically heterogeneous disease processes cannot be effectively addressed through uniform treatment approaches, a limitation that likely contributed to decades of inconclusive clinical trials despite promising preclinical data [146, 147]. Advances in multi‐omics technologies [18, 148], biomarker discovery [149, 150], and AI [75, 132, 151] are now enabling a more refined characterization of disease mechanisms, fostering the transition toward precision medicine frameworks.

**FIGURE 3:**
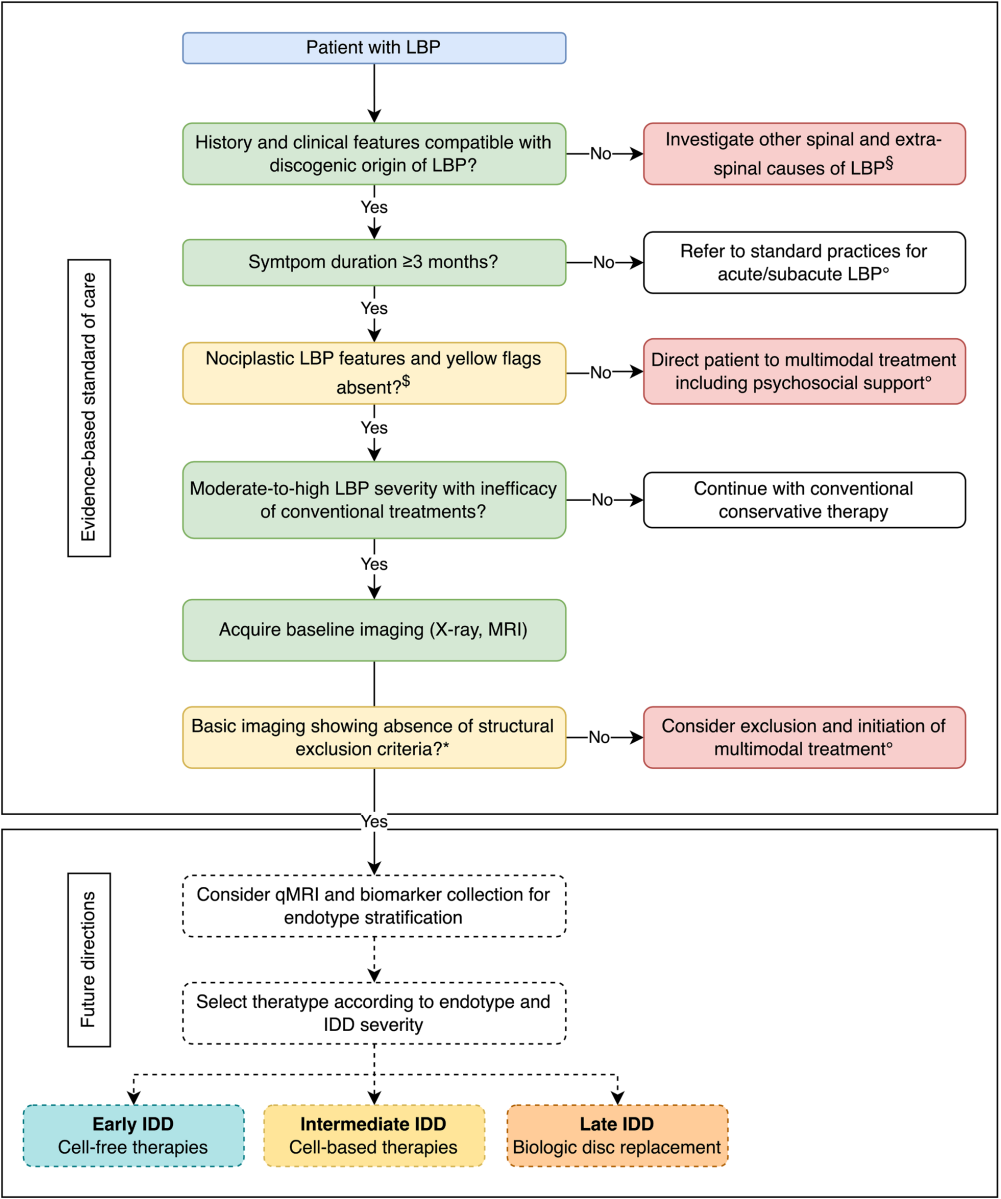
Clinical decision‐making algorithm for the identification and stratification of candidates for disc biologic therapies. Patients presenting with LBP are initially evaluated according to the evidence‐based standard of care, including clinical history, symptom duration, and non‐discogenic causes of LBP. Once nociplastic features and psychosocial “yellow flags” are ruled out, baseline imaging (i.e., X‐ray, MRI) is then performed to exclude structural contraindications. Patients meeting these criteria may be considered for advanced stratification approaches. Future directions include the integration of qMRI and biomarker profiling to define disease endotypes and guide personalized therapeutic selection. In this framework, treatment strategies are tailored to IDD severity, ranging from cell‐free approaches at early stages to cell‐based therapies at intermediate degeneration, and biologic disc replacement in advanced disease. ^§^Among extra‐spinal causes of LBP, red flags should be promptly recognized and addressed. °Management should follow the most recent international guideline recommendations [10, 12]. ^$^Screening for yellow flags should be performed using dedicated PROMs and application of the BACPAP consortium framework [46]. *Exclusion criteria include but are not limited to annular injury, significant endplate defects, segmental instability, previous fracture, etc. Abbreviations: IDD, intervertebral disc degeneration; LBP, low back pain; MRI, magnetic resonance imaging; ODI, Oswestry disability index; PROMs, patient‐reported outcome measures; qMRI, quantitative MRI; VAS, Visual Analogue scale.

Additionally, treatment selection should depend on whether the IVD and local milieu are compatible with the intended mechanism of action [62, 64]. Rather than broadly applying biologic therapies across heterogeneous IDD populations, future trials should define and screen for product‐specific eligibility criteria based on biological feasibility. Different classes of interventions are likely to require distinct local conditions to be (optimally) effective. Therapies aimed at enhancing ECM synthesis, such as cell‐based approaches [7] or growth factor delivery [152, 153], may depend on a local niche supportive of enhanced metabolic and biosynthetic activity [63, 64, 154]. In contrast, therapies targeting inflammatory signaling, such as PRP [155], may be more effective in discs with active inflammatory processes, while biomaterial‐based [156, 157] or gene therapy [158] strategies may require sufficient local or recruitable cells [159] and permissive biochemical conditions for remodeling and integration. Biomaterials represent an additional and highly versatile therapeutic category. Depending on their composition and functionalization, they may serve as structural scaffolds, drug delivery systems, modulators of the local microenvironment, or even as tissue replacements [88]. Although still largely confined to preclinical investigation, their potential applications may span across different stages of IDD. In early disease, biomaterials may be designed to enhance cellular activity and ECM synthesis; at intermediate stages, they may provide structural support and facilitate the retention and controlled release of biologic agents; whereas in advanced IDD, they may act as load‐bearing substitutes aimed at restoring disc height and biomechanical function [160]. A proposed operational algorithm for the identification and selection of eligible participants in clinical trials is illustrated in Figure 3.

However, current clinical trials rarely incorporate such product‐specific biological stratification criteria before patient enrolment. Even readily available morphological and biomechanical measures, including disc height preservation, annular and CEP integrity, and segmental stability, which may help identify stages of degeneration amenable to biologic intervention, are inconstantly incorporated as eligibility criteria. Failure to consider these context‐specific requirements may contribute to inconsistent or attenuated treatment effects, ultimately reinforcing the notion that a “one‐therapy‐fits‐all” strategy is unlikely to succeed in disc repair.

Overall, the integration of clinical, imaging, and biological data may enable the development of predictive indices of treatment feasibility or response, supporting more refined patient stratification prior to trial enrolment. Together, these strategies support a precision medicine framework in which each therapeutic modality is matched to a specific “Goldilocks zone” of disc biology, degeneration stage, and mechanical environment, thereby reducing heterogeneity and improving the likelihood of demonstrating meaningful clinical benefit.

### 7.3 Clinical Trial Design and Measuring What Matters

To improve the ability of clinical trials to detect true therapeutic effects, several methodological considerations should be addressed. A major challenge in injection‐based therapies for LBP is the consistently strong placebo response, which may mask or dilute treatment‐specific effects [90]. The use of appropriate control groups, including sham procedures where ethically and practically feasible, is therefore critical. An effective control should replicate the procedural aspects of the intervention without delivering the active therapeutic component, thereby isolating the biological effect of the treatment. However, in the context of intradiscal therapies, the use of sham injections warrants careful consideration, as disc puncture itself has been associated with acceleration of IDD [48, 49]. This raises important concerns regarding the validity and safety of such controls. Notably, the limited number of placebo‐controlled regenerative trials in LBP have reported substantial improvements in pain and disability within said sham groups [103, 161, 162], underscoring the magnitude of this effect. Accordingly, sample size calculations and power analyses should explicitly account for expected placebo responses to ensure adequate study design, sample size, and interpretation of efficacy outcomes.

In parallel, outcome assessment should move beyond reliance on subjective pain, disability, and quality of life measures, which are highly susceptible to placebo effects and inter‐individual variability. While appraising their clinical relevance, greater emphasis should also be placed on integrating objective and quantitative endpoints. Imaging‐based assessments should be systematically incorporated, quantified, and reported on, including parameters related to disc hydration, disc volume, and structural changes. Emerging techniques, including MRS and diffusion‐based imaging, may further provide insight into the molecular and metabolic state of the IVD. Functional imaging approaches, such as functional MRI, may offer additional opportunities to quantify and assess pain‐related neural activity and better characterize treatment effects at the central level [163, 164].

Complementary use of circulating or tissue‐derived biomarkers may enable assessment of biological responses through routine blood‐based analyses and serve as mechanistic indicators of treatment efficacy aligned with patient selection strategies [150, 165]. Furthermore, wearable technologies and smartphone‐based sensors have shown potential to objectively capture changes in physical activity and mobility, providing continuous, real‐world measures that complement traditional clinical outcomes [166, 167, 168]. Given their widespread use, integration of these tools into clinical trial design represents a potentially practical, scalable, and affordable approach to enhance objective outcome assessment.

Furthermore, trials should clearly define mechanistic targets, such as modulation of inflammation, restoration of ECM homeostasis, or inhibition of nociceptive signaling, and orient outcome measures accordingly. Such alignment across intervention, mechanism, and endpoints is essential to accurately interpret therapeutic efficacy and distinguish true biological effects from nonspecific or placebo‐driven responses, although broader implementation remains limited by the need for further validation and standardization of upcoming techniques and outcome parameters.

Finally, adequate follow‐up duration represents a critical aspect of trial design in this field. Regenerative processes within the IDD are inherently slow, and meaningful structural or biochemical changes may take years to manifest [63]. However, most clinical trials are limited to short‐ or mid‐term follow‐up (commonly 6–24 months) [161], potentially underestimating the true biological effects of the intervention (or demonstrating the clinical irrelevance of investigational treatments within a beneficial timeframe). Aligning follow‐up duration with the expected temporal dynamics of tissue repair would ideally enable a more accurate assessment of treatment efficacy and reduce the risk of prematurely concluding therapeutic failure. However, this must be balanced against the practical, economic, and ethical constraints associated with prolonging clinical trials beyond feasible and cost‐effective durations.

### 7.4 Navigating Translation: Regulatory and Practical Constraints

Translational and regulatory constraints should be considered early during trial design, as they directly impact feasibility and outcomes [97]. The choice between autologous and allogeneic products should be carefully considered, weighing the prolonged and costly manufacturing processes and patient‐specific variability associated with the former against the greater standardization and scalability of the latter, which can be sourced from screened, healthy donors and easily marketable [169, 170]. Therapeutic efficacy depends not only on product composition, but also on delivery method, dosing, and IVD microenvironment. Higher cell doses or concentrations of active factors do not necessarily improve outcomes and may even be harmful, highlighting the need for optimized, potentially patient‐specific dosing strategies [63, 64]. Single administration is generally preferable from both biological and practical perspectives, as repeated interventions increase complexity, costs, and regulatory burden, while also introducing patient risk and the potential to accelerate IDD progression by repeatedly puncturing the disc.

Regulatory classification necessitates evaluation of both the biologic and its delivery system, influencing procedural standardization, safety monitoring, and endpoint selection [98]. Increasing product complexity, such as the addition of biomaterials or multiple components, further amplifies manufacturing challenges and expands preclinical and clinical requirements to demonstrate safety and efficacy, while a clean and simple product likely presents a higher translational potential. In parallel, narrowing treatment to specific IDD phenotypes or biological niches may improve efficacy but reduce the eligible patient pool, with implications for trial recruitment and overall market scale and viability. Additionally, incorporating health economic considerations at early stages of product and trial design, such as durability of effect and reduced healthcare utilization, can help address these barriers and improve the likelihood of needed successful clinical translation. Eventually, harmonized and transparent reporting, together with efforts to minimize publication bias, is essential to avoid repeat investigations on ineffective products, which can lead to unnecessary investment and misallocation of attention and resources within healthcare systems and funding bodies.

## 8 The Road Ahead: Future Directions in Disc Biologic Therapies

At present, the field of disc regeneration is wandering in the dark, fearsome “valley of death” of clinical translation [77]. While the remarkable efforts of basic scientists have elucidated key aspects of IDD and laid the foundation for early translational attempts, several critical challenges persist. These include the need for more physiologically relevant in vitro systems, improved ex vivo models that accurately replicate the disc microenvironment and biomechanics, and in vivo models that better reflect the human condition.

Concurrently, clinical research must advance toward a more precise characterization of LBP, with the aim of aligning disc biology, clinical presentation, and stage of degeneration. The integration of clinical, imaging, and molecular data, supported by explainable and ethically robust AI frameworks, holds significant promise in this regard. Such approaches may ultimately reveal that biologic therapies for IDD are most effective within carefully selected patient subgroups, rather than broadly applicable across the heterogeneous chronic LBP population. In parallel, clinical trial methodology requires further refinement to account for the complexity of LBP phenotypes, particularly those influenced by psychosocial factors, which may introduce substantial confounding [90]. The adoption of stratified and adaptive trial designs may help mitigate these limitations and improve the detection of true treatment effects while reducing the risk of bias. Equally important is the need to enhance transparency, standardization, and methodological rigor in trial design and reporting. The implementation and widespread adoption of structured reporting frameworks, such as VERTEBRA [171], may facilitate reproducibility and enable more meaningful comparisons across studies.

Despite significant advances, key aspects of disc biology and treatment response remain poorly understood or assessed, underscoring the need for continued rigorous, collaborative research to translate biologic therapies into reliable clinical therapeutics. This decisive step may prompt researchers to reconsider the “regeneration dream”, as the complete restoration of the native IVD phenotype, irrespective of microenvironmental, structural, and neuroinflammatory changes occurring over decades, is likely unattainable, and its clinical relevance remains uncertain. Instead, the field should resignedly shift toward the concept of “repair”, intended as functional stabilization combined with targeted modulation of the biological processes underlying discogenic LBP.

## Author Contributions

**Daisuke Sakai:** writing – review and editing, supervision. **Luca Ambrosio:** conceptualization, investigation, writing – original draft, writing – review and editing, methodology, project administration. **Gianluca Vadalà:** writing – review and editing, supervision, funding acquisition. **Jordy Schol:** conceptualization, investigation, writing – original draft, writing – review and editing, methodology, project administration. **Clara Ruiz‐Fernandez:** investigation, writing – original draft, writing – review and editing. **Vincenzo Denaro:** writing – review and editing, supervision.

## Funding

This work was supported by NextGenerationEU, NRRP PNC‐E3‐2022‐23683269 PNC‐HLS‐TA (CUP code: F83C22002880001).

## Conflicts of Interest

Jordy Schol, Gianluca Vadalà, and Daisuke Sakai are Editorial Board members of JOR Spine and co‐authors of this article. To minimize bias, they were excluded from all editorial decision‐making related to the acceptance of this article for publication.

## Data Availability

Data sharing not applicable to this article as no datasets were generated or analysed during the current study.
